# Elevated Frequencies of Circulating Th22 Cell in Addition to Th17 Cell and Th17/Th1 Cell in Patients with Acute Coronary Syndrome

**DOI:** 10.1371/journal.pone.0071466

**Published:** 2013-12-03

**Authors:** Lei Zhang, Ting Wang, Xiao-qi Wang, Rui-zhi Du, Kai-ning Zhang, Xin-guang Liu, Dao-xin Ma, Shuang Yu, Guo-hai Su, Zhen-hua Li, Yu-qing Guan, Nai-li Du

**Affiliations:** 1 Department of Orthopedics, Shandong Provincial Qianfoshan Hospital, Shandong University, Jinan, China; 2 Department of Obstetrics, Shandong Provincial Qianfoshan Hospital, Shandong University, Jinan, China; 3 Department of Cardiology, Jinan Central Hospital Affiliated to Shandong University, Jinan, China; 4 Department of Cardiothoracic Surgery,Jinan Central Hospital Affiliated to Shandong University, Jinan, China; 5 Department of Hematology, Qilu Hospital, Shandong University, Jinan, China; INSERM- CNRS- Univ. Méditerranée, France

## Abstract

**Background:**

Atherosclerosis is a chronic inflammatory disease mediated by immune cells. Th22 cells are CD4^+^ T cells that secret IL-22 but not IL-17 or IFN-γ and are implicated in the pathogenesis of inflammatory disease. The roles of Th22 cells in the pathophysiologic procedures of acute coronary syndrome (ACS) remain unclear. The purpose of this study is to investigate the profile of Th22, Th17 and Th17/Th1 cells in ACS patients, including unstable angina (UA) and acute myocardial infarction (AMI) patients.

**Design and Methods:**

In this study, 26 AMI patients, 16 UA patients, 16 stable angina (SA) patients and 16 healthy controls were included. The frequencies of Th22, Th17 and Th17/Th1 cells in AMI, UA, SA patients and healthy controls were examined by flow cytometry. Plasma levels of IL-22, IL-17 and IFN-γ were measured by enzyme-linked immunosorbent assay (ELISA).

**Results:**

Th22, Th17 and Th17/Th1 cells were significantly increased in AMI and UA patients compared with SA patients and healthy controls. Moreover, plasma IL-22 level was significantly elevated in AMI and UA patients. In addition, Th22 cells correlated positively with IL-22 as well as Th17 cells in AMI and UA patients.

**Conclusion:**

Our findings showed increased frequencies of both Th22 and Th17 cells in ACS patients, which suggest that Th22 and Th17 cells may play a potential role in plaque destabilization and the development of ACS.

## Introduction

Atherosclerosis is a lipid-driven immune-inflammatory disease of arteries with the characteristic of multifocal plaque. Atherosclerosis may exert clinical effects by the dynamic development of plaque. With the plaques becoming obstructive, ischemic state may be induced. And the ischemia may lead to stable angina (SA). SA refers to the temporary clinical ischemia and hypoxia syndrome with typical presentations of chest discomfort and effort angina, which could be relieved with nitroglycerin or rest. Atherosclerotic plaque instability or rupture, which may cause thrombus formation in the coronary artery or coronary artery vasospasm, may trigger acute coronary syndromes (ACS). ACS represents a range of acute myocardial ischemic states that include unstable angina (UA) and acute myocardial infarction (AMI). UA is the clinical manifestation between SA and AMI. It is a worsening of angina symptoms caused by the formation of a thrombus that does not completely occlude the coronary artery and does not cause myocardial damage. AMI is similar to UA but more severe and with signs of myocardial ischemia on electrocardiogram.

Accumulating evidence has shown that atherosclerosis is a chronic inflammatory disease with macrophages and T-lymphocytes playing a critical role. Unstable plaque is characterized by an infiltrate of T cells and macrophages. Lesion resident macrophages may cause atherosclerotic plaque rupture by increasing secretion of matrix metalloproteinase. On activation, T cells secrete cytokines that regulate the activity of macrophages. Recently, it has been reported that CD4^+^ T-helper cells [1], [2] were involved in the development of atherosclerosis. ACS occurs as a consequence of coronary plaque rupture or plaque erosion, and changes in the functions of CD4^+^ T cells were found in patients with ACS [3], [4].

CD4^+^ T cells were traditionally divided into 2 subsets: Th1 cell and Th2 cell. Studies have shown that blockage of the Th1 pathway or knockout of IFN-γ or its receptor could inhibit the development of atherosclerosis [5]–[7]. Furthermore, it has also been demonstrated that T-bet deficiency, a critical transcription factor for Th1 cell differentiation, could reduce atherosclerosis development [8]. In recent years, a new IL-17-producing CD4^+^ T cell subset, termed as Th17 cells, has been identified [9]. Retinoid orphan nuclear receptor (ROR) is a key factor of Th17 subset differentiation [10], [11]. Th17 cell has been proven to play critical roles in inflammatory diseases, autoimmune diseases and graft-*versus*-host diseases by secreting IL-17A and other cytokines [12]–[14]. Several animal studies have revealed an important role for IL-17 in atherosclerosis [15]–[17]. However, several conflicting data have been demonstrated in ACS patients [18]–[20]. Thus, the role of IL-17 in atherosclerosis and ACS patients is still controversial.

More recently, a new human T helper subset, named as Th22 subset, has been identified. Th22 cell (CD4^+^IFN-γ^−^IL-17^−^IL-22^+^ T cell) is characterized by abundant production of IL-22 but not IL-17 or IFN-γ [21]–[23]. Similar to Th17 cell, Th22 cell expresses chemokine receptors CCR4 and CCR6. However, different from Th17 cell, Th22 cell can also express CCR10 [21], [22]. Naive T cells differentiate toward the Th22 phenotype in the presence of TNF-α and IL-6. In addition, aryl hydrocarbon receptor (AHR) is the key transcription factor of Th22 subset, while expression of transcription factor T-bet and RORγt (for Th1 and Th17, respectively) is low or undetected in this new CD4^+^ T cell subset. All of above evidences suggest that Th22 cell is an independent and terminally differentiated T subset. Several reports have demonstrated that Th22 cell may be implicated in chronic inflammatory diseases, including psoriasis [24], rheumatoid arthritis [25] and ankylosing spondylitis [26]. However, the implication of Th22 cells in other inflammatory diseases is far less understood.

IL-22, a critical effector cytokine of Th22 cell, is a member of IL-10 cytokine family. IL-22 can exert its effects via a heterodimeric transmembrane receptor complex consisting of IL-10R2 and IL-22R1 [27]. It has been deemed that IL-22 might play an important role in regulating inflammatory disease related inflammatory responses. Previous studies have shown that IL-22 was involved in several inflammatory and autoimmune diseases. However the results were controversial. Increased plasma IL-22 level was found in patients with psoriasis, rheumatoid arthritis and Crohn disease [28]. However, decreased plasma IL-22 level was shown in SLE patients [29]. Therefore, the precise pathophysiologic functions of IL-22 are still unclear.

The abnormality of T helper cell plays a critical role in the pathogenesis of atherosclerosis. However the underlying mechanisms of the abnormality of T helper cell in atherosclerosis have not been thoroughly elucidated. So far, the role of Th22 cells in atherosclerosis has not been reported. To determine their roles in the pathogenesis of atherosclerosis, we examined the frequencies of Th22 cells and levels of plasma IL-22 in peripheral blood of AMI, UA and SA patients, and assayed their correlations with the related clinical parameters in this study.

## Design and Methods

### Ethics statement

Enrollment took place between March, 2011 and July, 2012 in Jinan Central Hospital affiliated to Shandong University, China. Our research has been approved by the Medical Ethical Committee of Jinan Central Hospital affiliated to Shandong University. A written informed consent document has been obtained from each participant. The informed consent declared that remnant of patient's peripheral blood and healthy volunteer's peripheral blood was collected for scientific research on Th22 cells in the development of ACS.

### Patients and controls

A total of 74 subjects were recruited in this study. Subjects were classified into 4 groups: 1) The AMI group was composed of 16 men and 10 women with a mean age of 61.6±10.5 years, and inclusion criteria were as follows: chest pain lasting >30 min before enrollment and myocardial infarction confirmed by significant rise of creatin kinase MB and troponin I levels; 2) The UA group was composed of 10 men and 6 women with a mean age of 63.4±9.6 years, and inclusion criteria were as follows: chest pain with an accelerating pattern or prolonged duration (>20 min) or recurrent episodes at rest or with minimal effort with documented transient ST-segment elevation or ST-segment depression of 0.1 mV in at least two contiguous electrocardiograph leads. 3) The SA group was composed of 9 men and 7 women with a mean age of 62.5±10.1 years, and inclusion criteria were as follows: chest discomfort, including spreading to the left shoulder and arm, which could be relieved with nitroglycerin or rest. These patients had a down sloping or horizontal ST-segment depression<1 mm in an exercise test. 4) The HC (healthy control) group was composed of 10 men and 6 women with a mean age of 61.3±9.1 years, and subjects in this control group showed normal coronary arteries on angiography.

Not any patient was treated with immunosuppressive drugs and/or anti-inflammatory drugs. None had collagen disease, thromboembolism, renal failure, advanced liver disease, malignant disease or other inflammatory and autoimmune diseases (such as rheumatoid arthritis, psoriasis and systemic lupus erythematosus).

### Flow cytometric analysis

Before flow cytometric analysis, peripheral blood was collected and cultured under stimulation conditions. Briefly, heparinized peripheral whole blood (400 μl) in an equal volume of Roswell Park Memorial Institute 1640 medium were incubated for 4 h at 37°C with 5% CO_2_ in the presence of 25 ng/mL of phorbol myristate acetate (PMA), 1 μg/mL of ionomycin, and 1.7 μg/ml of Golgiplug (Monensin; all from Alexis Biochemicals, San Diego, CA, USA). PMA and ionomycin are pharmacological T-cell-activating agents that mimic signals generated by the T-cell receptor (TCR) complex and have the advantage of stimulating T cells of any antigen specificity. Monensin was used to block intracellular transport mechanisms, thereby leading to an accumulation of cytokines in the cells. After incubation, the cells were stained with PE-Cy5-conjugated anti-CD4 monoclonal antibodies (clone: RPA-T4, Cat: 45–0049–42) at room temperature in the dark for 20 min. After staining, the cells were fixed and permeabilized. Then the cells were stained with FITC-conjugated anti-interferon (IFN)-γ monoclonal antibodies (clone: 4S-BS, Cat: 11–7319–82), PE-conjugated anti-IL-17A monoclonal antibodies (clone: eBio64DEC17, Cat: 12–7179–42) and APC-conjugated anti-IL22 monoclonal antibodies (clone: 22URTI, Cat: 50–7229–42). All the antibodies were purchased from eBioscience, San Diego, CA, USA. Isotype controls were used to enable correct compensation and confirm antibody specificity. Stained cells were analyzed by flow cytometric analysis using a FACS cytometer equipped with CellQuest software (BD Bioscience PharMingen). Th22, Th17, Th1 and Th17/Th1 cells were defined as CD4^+^IFNγ^−^IL17^−^IL^−^22^+^, CD4^+^IFNγ^−^IL17^+^, CD4^+^IFNγ^+^ and CD4^+^IFNγ^+^IL17^+^ T cells respectively.

### Enzyme-linked immunosorbent assay (ELISA)

Peripheral blood was collected into tubes with heparin-anticoagulant. Plasma was obtained by centrifugation and stored at −80°C for determination of cytokines. Plasma IL-22 (Cat: BMS2047),IL-17 (Cat: BMS2017) and IFN-γ (Cat: BMS228) levels were determined with a quantitative sandwich enzyme immunoassay technique in accordance with the manufacturer's recommendations (eBioscience).

### Statistical analysis

Results were expressed as mean ± SD in the text and table. Statistical significance of the differences among the groups was determined by ANOVA, and difference between two groups was determined by Newman–Keuls multiple comparison test (*q* test). The Pearson correlation test was used for correlation analysis. All tests were performed by SPSS 17.0 system. P value less than 0.05 was considered statistically significant.

## Results

### Clinical characteristics

The clinical characteristics of subjects enrolled in this study were shown in Table 1. No significant differences were found in age, gender, number of diseased vessels, risk factors or medications among patients with AMI, UA and SA. Patients in AMI group exhibited higher levels of creatine kinase (CK), CK-MB, white blood cell count, cardiac troponin I and myoglobin than the other three groups (P<0.05; Table 1).

**Table 1:** Clinical characteristics of patients in the four groups.

Characteristics	AMI (n = 26)	UA (n = 16)	SA (n = 16)	HC (n = 16)
*Age*(*years*)	61.6±10.5	63.4±9.6	62.5±10.1	61.3±9.1
*Sex(male*:*female)*	16:10	10:6	9:7	10:6
*Numbers of* *diseased vessels*	1.9±0.8	1.7±0.9	1.8±0.6	0
*Current smoker* *n%*	13(50)	7(44)	8(50)	6(38)
*Current drinker* *n%*	9(35)	7(44)	6(38)	6(38)
*Hypertension* *n%*	16(62)	11(69)	10(63)	7(44)
*Diabetes mellitus* *n%*	6(23)	4(25)	5(31)	3(19)
*Dyslipidemia n%*	15(58)	7(44)	9(56)	6(38)
*WBC 10^9^/*	9.95±2.22*	6.59±1.81	6.47±2.29	6.38±1.32
*CK U/L*	1938±1491*	125±119	96.7±75.5	79.5±49.4
*CK-MB U/L*	176±155*	16.3±9.4	13.1±6.3	12.9±3.4
*cTnI ng/ml*	37.6±33.4*	0.3±0.4	0.12±0.22	0.07±0.22
*MYOG ng/ml*	307±127*	52.7±29.4	25.9±13.2	25.2±17.7
*Aspirin n%*	17(65)	10(63)	11(69)	2(13)
*Clopidogrel n%*	13(50)	7(44)	7(44)	0(0)
*ACEI/ARB n%*	14(54)	9(56)	8(50)	5(31)
*Beta-blockers* *n%*	15(58)	11(69)	10(63)	2(13)
*Calcium blockers* *n%*	5(19)	3(19)	3(19)	1(6)
*Statins n%*	15(58)	8(50)	9(56)	3(19)
*Nitrates n%*	16(62)	10(63)	9(56)	0(0)

Values are expressed as mean ± SD or number.

AMI: acute myocardial infarction; UA: unstable angina; SA: stable angina; HC: health control; WBC: white blood cell; CK: creatine kinase; cTnI: cardiac troponin I; MYOG: myoglobin.

ACEI: angiotensin-converting enzyme inhibitor; ARB: angiotensin II receptor blockers.

* P<0.05: AMI vs. UA/ SA/ HC.

### The percentages of circulating Th1 cells, Th22 cells and CD4^+^IFNγ^−^IL17^+^IL-22^+^ cells are increased in patients with AMI and UA

**Figure 1:**
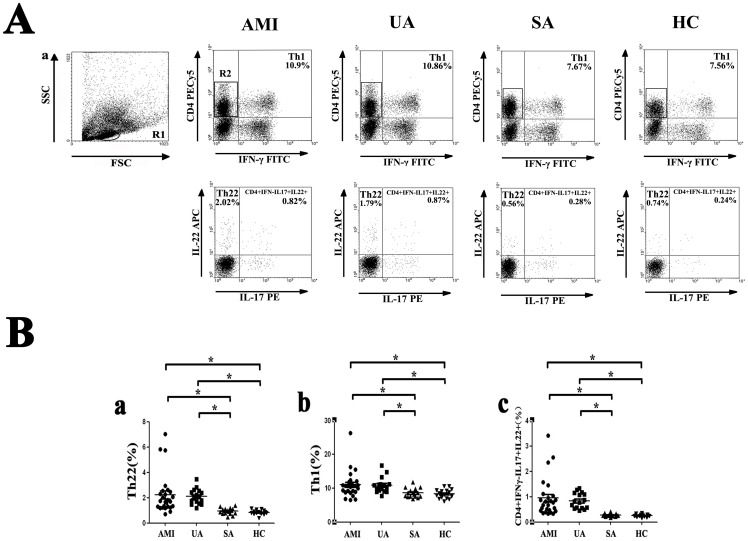
Flow cytometric analysis of Th1 cells, Th22 cells and CD4^+^IFNγ^−^IL17^+^IL-22^+^ cells. Peripheral blood from patients with AMI, UA, SA and HC subjects were stimulated with PMA, ionomycin and monensin for 4 h, and then stained with labeled antibodies as described in Methods. A, Gating strategies and representative flow cytometry dot plot results of each group. Lymphocytes were gated in R1 by forward and side scatter gating. These cells were analyzed for IFN-γ producing and CD4 expression T cells. CD4^+^ IFN-γ^−^ cells were gated in R2 and analyzed for IL-17 and IL-22 producing T cells. Numbers represent the percentage of cells in the quadrants. B, Comparison of the percentages of circulating Th22 cells (left panel, % of CD4^+^IFN-γ^−^ cells), Th1 cells (middle panel, % of total lymphocytes) and CD4^+^IFNγ^−^IL17^+^IL-22^+^ cells (right panel, % of CD4^+^IFN-γ^−^ cells) from AMI, UA, SA patients and healthy controls. (* = *P*<0.05, AMI and UA vs. SA and HC).

We examined the frequency of Th1 cells, Th22 cells and CD4^+^IFNγ^−^IL17^+^IL-22^+^ cells in peripheral blood by flow cytometry. Before flow cytometry analysis, cells in peripheral blood were activated by PMA/ ionomycin in vitro. Representative flow cytometry results were shown in Fig. 1A and quantitative results were shown in Fig. 1B. By forward and side scatter gating, lymphocytes were identified and gated in region R1. These lymphocytes were used for the analysis of IFN-γ producing and CD4 expression T cells. Cells gated in region R2 were CD4^+^ IFN-γ^−^ cells. These subsets were analyzed for IL-17 and IL-22 producing T cells. The percentage of Th22 cells (CD4^+^IFN-γ^−^IL22^+^IL17^−^ cells) in CD4^+^IFN-γ^−^ cells was notably increased in AMI (2.23±1.58%) and UA (2.09±0.60%) patients compared to SA patients (0.93±0.26%) or healthy controls (0.84±0.18%) (AMI vs. UA, P = 0.648; AMI vs. SA, P<0.001; AMI vs. HC, P<0.001; UA vs. SA, P = 0.002; UA vs. HC, P = 0.001; SA vs. HC, P = 0.793) (Fig. 1B).

Consistent with Th22 cells, the percentage of Th1 cells (CD4^+^IFN-γ^+^ cells) in total lymphocytes was significantly increased in AMI (11.08±3.94%) and UA (10.85±2.33%) patients compared to SA patients (8.61±1.37%) or healthy controls (8.35±1.36%) (AMI vs. UA, P = 0.789; AMI vs. SA, P = 0.006; AMI vs. HC, P = 0.002; UA vs. SA, P = 0.024; UA vs. HC, P = 0.012; SA vs. HC, P = 0.786) (Fig. 1B). Moreover, to check whether Th17 cells could develop into IL-22 producing Th17 cells in the local inflammation environment of atherosclerosis, we analyzed CD4^+^IFN-γ^−^IL-17^+^IL-22^+^ cells separately. The percentage of CD4^+^IFNγ^−^IL17^+^IL-22^+^ T cells in CD4^+^IFN-γ^−^ cells was also significantly elevated in AMI (0.95±0.76%) and UA (0.84±0.30%) patients compared to SA patients (0.28±0.06%) or healthy controls (0.25±0.05%) (AMI vs. UA, P = 0.451; AMI vs. SA, P<0.001; AMI vs. HC, P<0.001; UA vs. SA, P = 0.001; UA vs. HC, P = 0.001; SA vs. HC, P = 0.889) (Fig. 1B).

### The percentages of circulating Th17 cells and Th17/ Th1 cells are increased in AMI and UA patients

**Figure 2:**
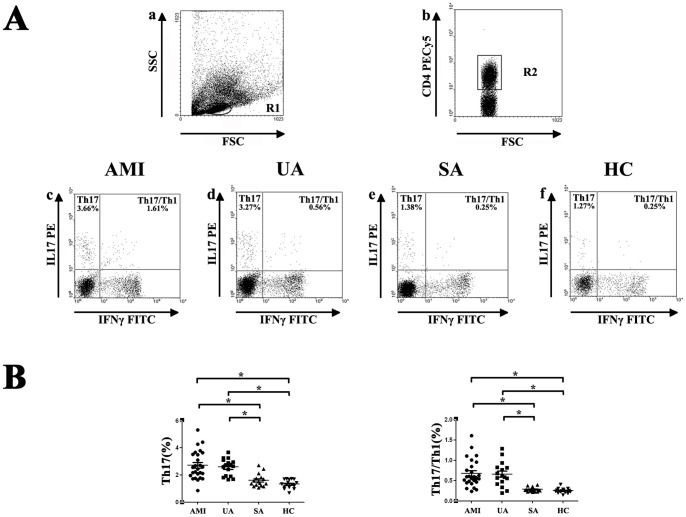
Flow cytometric analysis of Th17 cells and Th17/ Th1 cells. Peripheral blood from patients with AMI, UA, SA and HC subjects were stimulated with PMA, ionomycin and monensin for 4^+^ cells were gated in R2 and analyzed for IL-17 and IFN-γ producing T cells. Numbers represent the percentage of cells in the quadrants. B, Comparison of the percentages of Th17 cells and Th17/Th1 cells in CD4^+^ cells from AMI, UA, SA patients and healthy controls. (* = *P*<0.05, AMI and UA vs. SA and HC).

The frequency of Th17 cells and Th17/Th1 cells was also examined in this study. Similarly, cells in peripheral blood were activated by PMA/ ionomycin in vitro before flow cytometry analysis. By forward and side scatter gating, lymphocytes were gated in region R1 and analyzed for CD4 expression T cells. Cells gated in region R2 were CD4^+^ cells and analyzed for IL-17 and IFN-γ producing T cells. Representative flow cytometry dot-plot results were shown in Fig. 2A and quantitative results were shown in Fig. 2B. Compared with SA patients (1.60±0.48%) and healthy controls (1.34±0.32%), the percentages of Th17 cells were significantly increased in AMI (2.72±1.04%) and UA (2.59±0.62%) patients (AMI vs. UA, P = 0.585; AMI vs. SA, P<0.001; AMI vs. HC, P<0.001; UA vs. SA, P<0.001; UA vs. HC, P<0.001; SA vs. HC, P = 0.306) (Fig. 2B). Consistently, we also found that the percentages of Th17/Th1 cells were notably elevated in AMI (0.68±0.34%) and UA (0.66±0.30%) patients compared with SA patients (0.28±0.05%) and healthy controls (0.25±0.06%) (AMI vs. UA, P = 0.813; AMI vs. SA, P<0.001; AMI vs. HC, P<0.001; UA vs. SA, P<0.001; UA vs. HC, P<0.001; SA vs. HC, P = 0.743) (Fig. 2B).

### Correlation between Th22, Th17 and Th1 cells in AMI and UA patients

**Figure 3:**
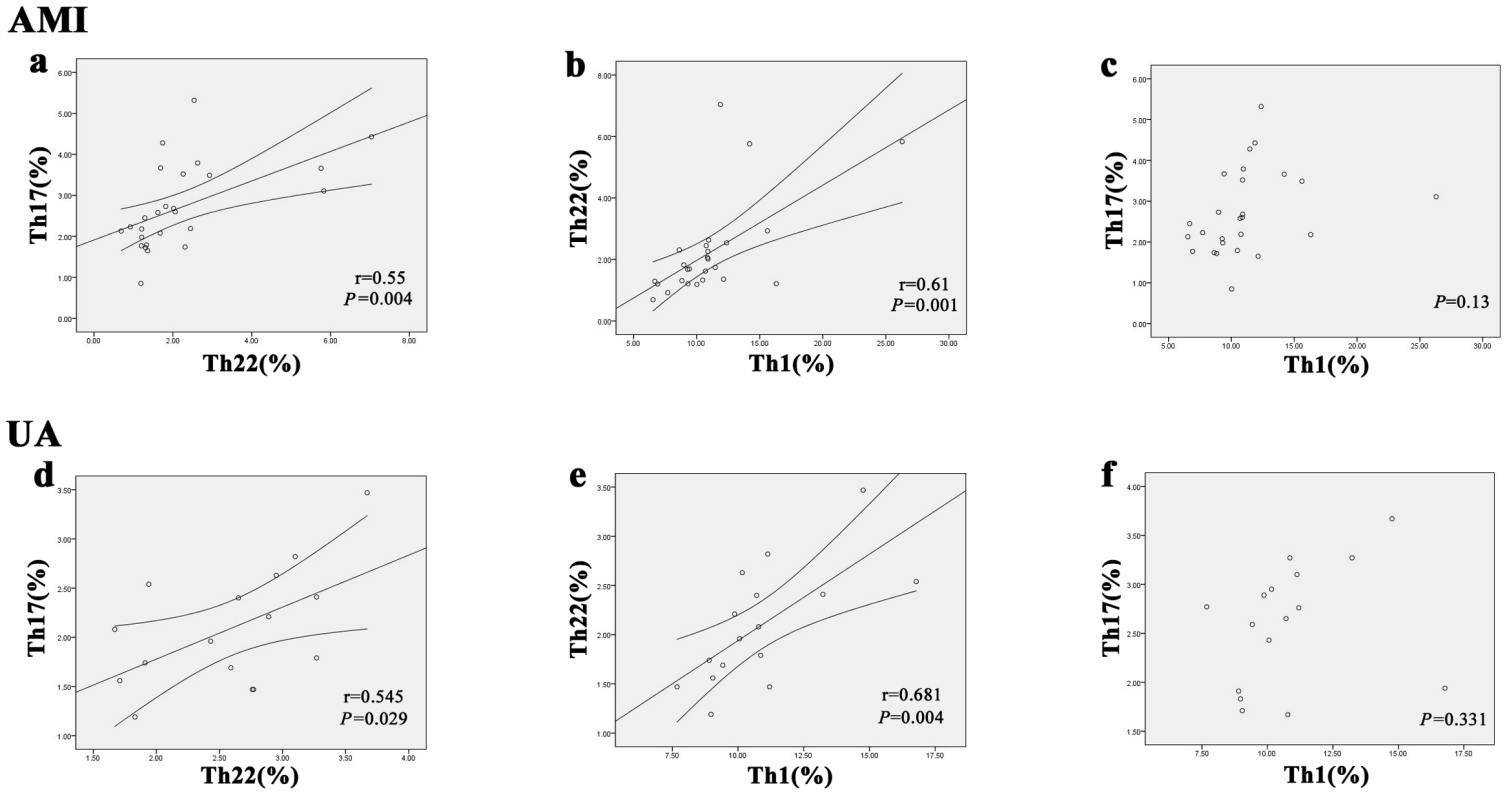
Correlation analysis between the percentages of Th17 cells, T22 cells and Th1 cells in AMI and UA patients. a and d, Correlation between percentages of Th17 cells and T22 cells in AMI (r = 0.55, P = 0.004) and UA (r = 0.545, P = 0.029) patients. b and e, Correlation between percentages of Th22 cells and T1 cells in AMI (r = 0.61, P = 0.001) and UA (r = 0.681, P = 0.004) patients. c and f, Correlation between percentages of Th17 cells and T1 cells in AMI (p = 0.13) and UA (p = 0.331) patients.

In AMI patients, a positive correlation was observed between Th22 cells and Th17 cells (r = 0.55, P = 0.004) (Fig. 3a). Similarly, a positive correlation was also found between Th22 cells and Th17 cells (r = 0.545, P = 0.029) (Fig. 3d) in UA patients. Moreover, there was a positive correlation between Th22 cells and Th1 cells in patients with AMI (r = 0.61, P = 0.001) (Fig. 3b) and UA (r = 0.681, P = 0.004) (Fig. 3e).

Nevertheless, Th1 cells did not show a significant correlation with Th17 cells in both AMI patients (P = 0.13) (Fig. 3c) and UA patients (r = 0.46, P = 0.331) (Fig. 3f).

### Plasma concentrations of IL-22, IL-17 and IFN-γ among the four groups

**Figure 4:**
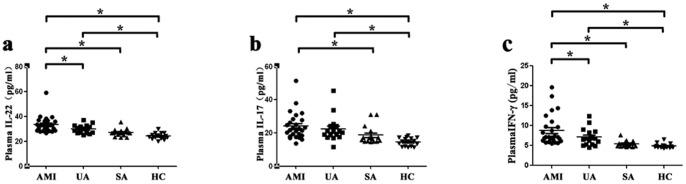
Levels of IL-22, IL-17 and IFN-γ in plasma of non-stimulated peripheral blood from AMI patients, UA patients, SA patients and healthy controls. Plasma IL-22, IL-17 and IFN-γ levels were detected by ELISA assay. a, Levels of plasma IL-22 in AMI, UA, SA and HC subjects. b, Levels of plasma IL-17 in AMI, UA, SA and HC subjects. c, Levels of plasma IFN-γ in AMI, UA, SA and HC subjects. (* = *P*<0.05, AMI and UA vs. SA and HC).

Plasma concentrations of IL-22, IL-17 and IFN-γ were examined by ELISA in this study. The level of plasma IL-22 was significantly elevated in AMI patients (33.09±6.53 pg/ml) compared with UA patients [(29.86±3.49 pg/ml), P = 0.02], SA patients [(26.96±3.09 pg/ml), P<0.001] and healthy controls [(24.16±2.46 pg/ml), P<0.001] (Fig. 4a). In addition, the level of plasma IL-22 was significantly elevated in UA patients (29.86±3.49 pg/ml) compared to healthy controls [(24.16±2.46 pg/ml), P = 0.001]. However, there was no significant difference between SA patients (26.96±3.09 pg/ml) and UA patients [(29.86±3.49 pg/ml), P = 0.078] or healthy controls [(24.16±2.46 pg/ml), P = 0.09].

The level of plasma IL-17 was significantly elevated in AMI patients (24.18±8.07 pg/ml) compared with SA patients [(18.70±5.49 pg/ml), P = 0.012] and healthy controls [(24.16±2.46 pg/ml), P<0.001] (Fig. 4b). Consistent with the result of IL-22, the level of plasma IL-17 was significantly elevated in UA patients (22.67±7.97 pg/ml) compared to healthy controls [(24.16±2.46 pg/ml), P = 0.002], and no significant difference was found between AMI patients (24.18±8.07 pg/ml) and UA patients [(22.67±7.97 pg/ml), P = 0.383].

The level of plasma IFN-γ was significantly elevated in AMI patients (8.81±3.81 pg/ml) compared with UA patients [(7.09±2.21 pg/ml), P = 0.037], SA patients [(5.40±0.79 pg/ml), P<0.001] and healthy controls [(4.89±0.58 pg/ml), P<0.001] (Fig. 4c). Moreover,the level of plasma IFN-γ was significantly elevated in UA patients (7.09±2.21 pg/ml) compared to healthy controls [(4.89±0.58 pg/ml), P = 0.016].

### Correlation between percentages of Th22, Th17 and Th1 cells and levels of IL-22, IL-17 and IFN-γ cytokines in AMI and UA patients

**Figure 5:**
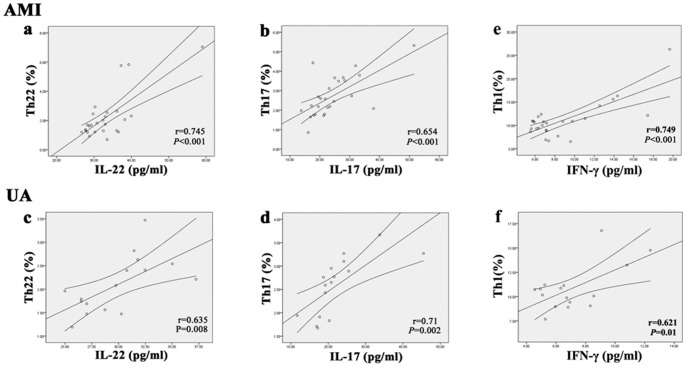
Correlation analysis between T cells and related cytokines in AMI and UA patients. a and c, Correlation between percentages of Th22 cells and plasma IL-22 levels in AMI (r = 0.745, P<0.001) and UA patients (r = 0.635, P = 0.008). b and d, Correlation between percentages of Th17 cells and plasma IL-17 levels in AMI (r = 0.654, P<0.001) and UA (r = 0.71, P = 0.002) patients. e and f, Correlation between percentages of Th1 cells and plasma IFN-γ levels in AMI (r = 0.749, P<0.001) and UA (r = 0.621, P = 0.01) patients.

There was a positive correlation between Th22 cells and plasma levels of IL-22 in AMI (r = 0.745, P<0.001; Fig. 5a) and UA (r = 0.635, P = 0.008; Fig. 5c) patients. Moreover, positive correlations were also found between IL-17 plasma levels and Th17 cells in AMI (r = 0.654, P<0.001; Fig. 5b) and UA (r = 0.710, P = 0.002; Fig. 5d) patients. Similarly, a positive correlation was observed between IFN-γ plasma levels and Th1 cells in AMI (P<0.001, r = 0.749; Fig. 5e) and UA (P = 0.01, r = 0.621; Fig. 5f) patients. However, there was no significant correlation between plasma IL-22 levels and percentages of Th17 cells in AMI (p = 0.134) and UA patients (p = 0.262) (data not shown).

## Discussion

It has been demonstrated that inflammation contributed to the onset and development of atherosclerosis. Immune cells, particularly macrophages and T helper cells, are involved in the atherogenesis. On activation, T cells secrete cytokines that regulate the activity of macrophages. In recent years, Th22 subset, the newest member of the T helper subsets, is identified. It is characterized by its ability to secret IL-22 and TNF-α but not IL-17 or IFN-γ. Researches have demonstrated that Th22 cells were implicated in the pathogenesis of a variety of inflammatory diseases and autoimmune diseases. For example, elevated Th22 cell levels were detected in peripheral blood of patients with psoriasis [24], suggesting the implication of Th22 cells in the chronic inflammatory skin disorder. Furthermore, Th22 cells were involved in the pathogenesis of both RA and AS, which were two types of inflammatory arthritis [25], [26]. These researches indicate that Th22 cells may have pathogenic effects on inflammatory diseases. To assess whether Th22 cells is involved in the development of atherosclerosis, we examined the expression of Th22 cells in the peripheral blood of patients with AMI, UA, SA and of subjects in HC. We found that the numbers of Th22 cells were significantly increased in AMI and UA patients in comparison to SA and HC subjects. Thus, our data suggested that Th22 cells may be involved in the inflammatory process in plaque destabilization. However, previous studies on Th22 cells in the process of infection, inflammation and autoimmunity suggest that Th22 cells may play a biphasic role depending on the focal microenvironment [22], [30], [31]. Thus, the underlying mechanisms of Th22 cells in local plaque and in the pathogenesis of ACS are unclear and need to be further investigated.

The proinflammatory effects of Th22 in the development of atherosclerosis may be dependent on the synergistical effects of IL-22 and TNF-α, which are the main effective cytokines of Th22 cells. In the present study, plasma IL-22 level was measured by ELISA assay. Similar to Th22 cells, patients with AMI exhibited remarkable rise in the level of plasma IL-22 compared to UA, SA and HC groups. However, no significant difference of plasma IL-22 level was observed between UA patients and SA patients in this study.

As a proinflammatory cytokine, IL-22 could increase inflammatory cytokines and chemokines in colonic subepithelial myofibroblasts [32] and induce the production of MCP-1 in synovial fibroblasts [33]. Moreover, it has been observed [34] that the expression of S100A7, S100A8, and S100A9, which were a group of pro-inflammatory molecules belonging to the S100 family of calcium-binding proteins, were up-regulated in the presence of IL-22. Hanawa et al reported that IL-22 could interact with fibroblasts, smooth muscle cells, and endothelial cells in the rat experimental autoimmune myocarditis (EAM) study [35]. IL-22 was considered to induce acute phase response by inducing the production of acute-phase protein [36]. According to above-mentioned reports, we speculated that Th22 cells may participate in the process of ACS by IL-22 secretion. In addition, our study also showed the positive correlation between peripheral Th22 cells and plasma IL-22 levels in both AMI patients and UA patients, which further suggest that Th22 cells was the main IL-22 secreting T cells in peripheral blood of AMI and UA patients. As another representative effector molecule of Th22 cells, TNF-α was considered to potently promote atherosclerosis in mice and humans [37]–[39]. And it has been reported that RAF1 deficiency could ameliorate atherosclerosis in mice [40]. Consistent with our study, Rajappa et al observed that AMI and UA patients exhibited higher serum levels of TNF-α compared to SA and HC subjects [41]. In addition, elevated levels of TNF-α and IL-17 had a synergistic effect on promoting the development of atherosclerosis [42]. The effect of TNF-α on atherosclerosis further proved that Th22 cells may be one of the most important T cells involved in atherosclerosis.

In the present study, we also demonstrated that ACS patients exhibited a marked elevation in the percentage of peripheral Th17 cells when compared to SA and HC subjects. This result was consistent with the data of Cheng et al [19]. Th17 cells could stimulate epithelial cells, endothelial cells and fibroblastic cells to secret proinflammatory cytokines and chemokines. Furthermore, Th17 cells were reported to be implicated in the activation and aggregation of macrophages in plaque. Macrophages could release matrix metalloproteases, leading to plaque destabilization. IL-17 is the main effective cytokine of Th17 cells, which exhibits proinflammatory properties and plays important roles in the activation, recruitment and migration of neutrophils [43]. And it has been reported that the elevated IL-17 level may promote early plaque formation in mice [44]. Therefore, IL-17 might mediate the occurrence of ACS. In our study, we found that AMI patients exhibited a remarkable rise in the level of plasma IL-17 when compared to SA and HC subjects. However, no significant differences in plasma IL-17 were found between AMI and UA patients. Moreover, the differences were also not found in UA and SA patients. This result was different from the data reported by Li et al. They observed significant difference in the level of IL-17 between UA and SA [18]. This inconsistence may be caused by the number of patients used or the difference of IL-17 level in plasma and in serum. Our results indicate that IL-17 was an important pathogenic cytokine in ACS. Collectively, the roles of IL-17 in atherosclerosis are complicated and need further investigation. Furthermore, positive correlation between Th17 cells and IL-17 was also shown in AMI patients and UA patients in our study, which suggest that Th17 cell was the main source of IL-17 in peripheral blood.

Th1 cells have also been shown to be involved in the development of atherosclerosis. In accordance with the data reported by Zhao et al. [45] and Methe et al. [3], our study also demonstrated remarkable rises in the percentage of peripheral Th1 cells in AMI and UA patients. Zhao et al have also shown the enhanced Th1-related mRNA levels, including T-bet and IFN-γ. Moreover, positive correlations were observed between Th22 cells and Th1 cells in patients with AMI and UA in the current study. In addition, this relationship also existed between Th22 cells and Th17 cells in patients with AMI and UA. However, such relationships were not found between Th17 cells and Th1 cells in AMI and UA patients in our study. These positive correlations suggest that these T cell subsets may play a synergistic role in the development of AMI and UA. The differentiation of Th22, Th1 and Th17 cells may be driven in an influential manner in ACS patients. IL-6 is not only required for IL-17 induction from naïve T cells but also can promote the expression of IL-22 [46]. In addition, IL-23 is essential for human Th17 differentiation, and IL-23 treatment can induce IL-22 production. These associations between cytokines might contribute to the positive correlation between Th22 cells and Th17 cells in our study. Detailed mechanisms need further investigation. Moreover, plasma levels of IFN-γ, which is the main effector cytokine of Th1 cells, were also significantly elevated in AMI patients compared with subjects in UA, SA and HC group. Th1 cells were involved in the development of atherosclerosis through secreting IFN-γ. IFN-γ was a potent activator for macrophages and vascular endothelial cells, which could activate macrophages and promote their activity. It was demonstrated that IFN-γ played a key role in the formation of foam cells and development of atherosclerotic plaque [47]. In addition, the elevated frequencies of Th1 cells were positively correlated with the increased plasma levels of IFN-γ in AMI patients. This result further indicates that Th1 cells played their role in the development of atherosclerosis through secreting IFN-γ.

It is not unusual to encounter Th17/Th1 cells. Th17/Th1 cells have been previously reported in many researches, suggesting a possible conversion between Th17 cells and Th1 cells [48]–[50]. It has been demonstrated that Th17 cells could convert into Th1-like cells and that these Th1-like cells were more pathogenic than Th17 cells in diabetes mouse model [49]. In addition, Th17/Th1 cells activation has been demonstrated to be associated with thin fibrous cab and increased macrophages in animal model with atherosclerotic plaque [51]. In accordance with Zhao et al. study [45], our study showed that the frequency of Th17/Th1 cells was significantly increased in AMI and UA patients in comparison to SA and HC subjects. Our results further indicate that Th17/Th1 cells may be implicated in the development of ACS. Moreover, our study demonstrated for the first time that compared with the subjects of SA and HC, patients with AMI and UA exhibited a remarkable rise in peripheral percentages of CD4^+^IFNγ^−^IL17^+^IL-22^+^ T cells, which suggest that CD4^+^IFNγ^−^IL17^+^IL-22^+^ T cells may also be involved in the process of ACS. Although IL-22 producing Th17 cells were not an independent cell subset, they were analyzed separately in many studies. For example, Colin et al [52] found elevated percentages of IL-17^+^ and IL-22^+^CD4^+^ T cells in PBMCs from patients with early rheumatoid arthritis (RA). Similarly, Zhang et al [26] detected increased percentages of CD4^+^IFN-γ^−^IL-17^+^IL-22^+^ cells in peripheral blood from ankylosing spondylitis (AS) and RA patients. In experimental autoimmune encephalomyelitis (EAE), IL-22 was postulate to contribute to the pathogenic function of Th17 cells and IL-22 producing Th17 cells were the highly pathogenic population of self-reactive T cells [53]. Therefore, Th17 cells tend to secret IL-22 and develop into IL-22 producing Th17 cells in the local immune environment of chronic inflammatory diseases and autoimmune diseases. And this tendency may be a common immunologic characteristic. Thus our results further indicate that Th17 cells in the local inflammation environment of atherosclerosis could develop into IL-22 producing Th17 cells. Taken together, we suppose that increase of CD4^+^IFN-γ^−^IL-17^+^IL-22^+^ cells as well as elevation of Th17/Th1 cells were immunologic characteristics of ACS.

In summary, our data showed that the frequencies of circulating Th22 cells, Th17 cells and Th17/Th1 cells and the levels of plasma IL-22, IL-17 and IFN-γ were significantly increased in patients with ACS. The increasing percentages of T helper subsets and the elevation of related cytokines may contribute to the development of atherosclerotic plaque instability and pathogenesis of ACS. Further research should be performed to identify the precise mechanism of the implication of Th22 cells in plaque procession and destabilization in atherosclerosis. Th22 and Th17 cells as well as related cytokines may be considered as new targets for treatment of atherosclerosis and ACS.
